# Taxonomic and ecological discrimination of Fagaceae species based on internal transcribed spacer polymerase chain reaction–restriction fragment length polymorphism

**DOI:** 10.1093/aobpla/plu079

**Published:** 2014-11-26

**Authors:** João Paulo Coutinho, Ana Carvalho, José Lima-Brito

**Affiliations:** Institute of Biotechnology and Bioengineering (IBB), Centre of Genomics and Biotechnology (CGB), University of Tras-os-Montes andAlto Douro, 5001-801 Vila Real, Portugal

**Keywords:** *Castanea*, *Fagus*, internal transcribed spacer (ITS), *Quercus*, restriction fragment length polymorphism (RFLP).

## Abstract

The internal transcribed spacer (ITS) of ribosomal DNA has been used to confirm taxonomic classifications and define phylogenies in several plant species following sequencing or polymerase chain reaction–restriction fragment length polymorphism (PCR–RFLP) techniques. In this study, co-dominant ITS PCR–RFLP molecular markers were produced in 30 Fagaceae individuals belonging to the *Castanea*, *Fagus* and *Quercus* genera in order to assess the potential of this technique for taxonomic discrimination and determination of phylogenies. The complete ITS region (ITS1-5.8S rRNA-ITS2) was amplified in most of the Fagaceae individuals as a single fragment of ∼700 bp. The ITS amplified products were digested with nine restriction enzymes, but only four (HaeIII, HpaII, TaqI and Sau96I) produced polymorphic/discriminative patterns. The total expected heterozygosity (*H*_E_) was 20.31 % and the gene diversity (*I*), 32.97 %. The ITS polymorphism was higher within the *Quercus* genus (85.3 %). The ITS PCR–RFLP markers clustered the Fagaceae species according to genus or infrageneric group (in the case of *Quercus* sp. individuals). Five oaks did not cluster in line with the adopted infrageneric classification, but three of these were grouped according to their actual ecological distributions. The ITS PCR–RFLP markers indicated their potential for phylogenetic studies since all Fagaceae individuals were discriminated according to genus, and most of the oaks were clustered according to infrageneric group or ecological area.

## Abstract (precis)

In 30 Fagaceae individuals from the *Castanea*, *Fagus* and *Quercus* genera, the internal transcribed spacer region (ITS) of the ribosomal DNA (rDNA) was amplified and digested with four restriction enzymes (*Hae*III, *Hpa*II, *Sau*96I, and *Taq*I), producing ITS PCR-RFLP markers. This technique allowed the discrimination of the Fagaceae species according to genus, infrageneric group and ecological area. This study constitutes the first application of the alternative co-dominant marker system ITS PCR-RFLP in Fagaceae species and proved to be highly suitable for taxonomic and ecological discrimination, constituting a useful molecular tool for taxonomists and forestry researchers.

## Introduction

Ribosomal DNA (rDNA) is a nuclear multigene family with copies arranged in tandem arrays within the nucleolar organizer regions (NORs) that form the secondary chromosomal constriction, with the centromere being the primary constriction (McClintock 1934; Navashin 1934). Each rDNA unit is composed of the conserved coding regions of the 18S-5.8S-26S rRNA genes, including the variable non-coding intergenic spacer (IGS). The IGS itself consists of a non-transcribed spacer region containing motifs that are designated as sub-repeats and is flanked by external transcribed spacers. The 5.8S rRNA gene is flanked by two non-coding sequences referred to as the internal transcribed spacers (ITS), ITS1 and ITS2, constituting the entire ITS region. Despite being fast-evolving regions, the rDNA spacers experience concerted evolution, and low diversity is expected within and among the rDNA loci. Such sequence uniformity allows the numerous rDNA copies in a genome to be treated as a single gene. Furthermore, the biparental inheritance, universality and orthology/paralogy of the rDNA sequences allow the use of ITS in taxonomic inferences and phylogenetics among diverged taxa (Baldwin *et al.* 1995).

As an alternative to sequencing or studies involving the laborious Southern blotting technique (Carvalho *et al.* 2010, 2013), the polymerase chain reaction–restriction fragment length polymorphism (PCR–RFLP) technique has been applied to the ITS or IGS rDNA spacers, resulting in co-dominant molecular marker systems that have proved useful for the detection of length variants, establishment of genetic relationships and assessment of genetic diversity in different plant species (Quijada *et al.* 1998; Nwakanma *et al.* 2003; Nalini *et al.* 2007; Saini *et al.* 2008; Carvalho *et al.* 2009, 2011*a*, *b*; Lima-Brito *et al.* 2011). Due to their repetitive nature, the spacers tend to be more variable than the coding regions of the rDNA. In plants, the longer ITS1 seems to evolve more rapidly than ITS2 (in *Pinus* sp.; Quijada *et al.* 1998); it also exhibits higher levels of informative phylogenetic information than ITS2 (in oaks; Bellarosa *et al.* 2005). Nevertheless, the sequence conservation of rDNA loci has led to an intra-specific degree of polymorphism with <5 % of sequence divergence (Mayol and Rosselló 2001; Bellarosa *et al.* 2005). Such slow and consistent mutational behaviour might have increased the utility of ITS in phylogenetics. Despite the concerted evolution of rDNA, intra-individual variability may be found in Fagaceae species when sequence homogenization among paralogues has not yet achieved completion (Mayol and Rosselló 2001; Muir *et al.* 2001). However, the identification of paralogues in an individual does not always seem to imply the presence of pseudogenes (Denk *et al.* 2002).

Several studies have resorted to ITS sequencing for taxonomic clarification in this family, particularly in the assessment of phylogenetic relations in *Quercus* (Manos *et al.* 1999, 2001; Bellarosa *et al.* 2005; Casas *et al.* 2007; Denk and Grimm 2010), *Castanea* (Manos *et al.* 2001) and *Fagus* (Denk *et al.* 2002). The fact that the RFLP technique is rapid and able to analyse a large number of samples simultaneously has made it useful for studying the chloroplastidial DNA (cpDNA) or mitochondrial DNA in the *Quercus* genus, and has enabled the following: evaluation of the cpDNA variation of *Quercus suber* L. in the Mediterranean basin (Lumaret *et al.* 2005); study of the level of cytoplasmic exchange between *Q. suber* and *Q. ilex* L. in Morocco (Belahbib *et al.* 2001); investigation of the dynamics of the spread of *Q. robur* L. and *Q. petraea* (Mattuschka) Liebl. throughout England (Cottrell *et al.* 2002); retracing of the recolonization routes of white oaks in the Alps (Csaikl *et al.* 2002); and inference of the structure of the *Q. affinis* Scheidweiler— *Q. laurina* Humboldt complex in Mexico (González-Rodriguez *et al.* 2004).

Subjecting the amplified ITS1-5.8S-ITS2 region to the RFLP technique may allow the evaluation of the ITS polymorphism. Instead of searching for dissimilarities in a nucleotide sequence, the RFLP methodology identifies only the differences associated with the presence/absence of endonuclease recognition sites due to mutations, insertions or deletions. As a result, only highly prevalent ITS differences present in the large rDNA multigene family of an organism will allow the development of a visible band in an agarose gel, thus producing a specific band pattern.

In the current study, our aim was to evaluate the ITS polymorphism in individuals from the genera *Castanea* Mill., *Fagus* Tourn and *Quercus* L. using PCR–RFLP performed with nine restriction enzymes. We also aimed to investigate the potential of the ITS PCR–RFLP markers for taxonomic discrimination and determination of phylogenies, with a particular focus on the genus *Quercus*.

## Methods

### Plant material

The plant material used in this study is listed in Table 1 and comprised 30 species belonging to the Fagaceae family: 3 chestnuts (*Castanea* Mill.), 3 beeches (*Fagus* Tourn) and 24 oaks (*Quercus* L.).

**Table 1.** Taxonomical classification, ecological distribution, seed origin and voucher numbers of the Fagaceae individuals studied (HVR designation is in accordance with Index Herbariorum codes; Thiers 2010). *Seeds were provided by the following botanical gardens: Arboretumul Simeria, Hunedoara, Romania; Botanical Garden of the University of Tras-os-Montes and Alto Douro, Vila Real, Portugal; Botanischer Garten der Universität Tübingen, Tübingen, Germany; Botanischer Garten der Westfälische Wilhelms, Universität Münster, Germany; Botanischer Garten Duisburg, Duisburg, Germany; Dubrava Arboretum, Kaunas, Lithuania; Hortus Botanicus Tallinnensis, Tallinn, Estonia; Hortus Botanicus, Universitatis Latviensis, Riga, Latvia; Jardin Botanique de la Ville de Lyon, Lyon, France; Kamigamo Experimental Station, Kyoto University, Kyoto, Japan; Kanagawa Prefectural Ofuna Botanical Garden, Okamoto, Kamakura, Kanagawa, Japão; Ökologisch Botanischer Garten, Universität Bayreuth, Bayreuth, Germany; Orto Botanico, Universita' di Siena, Siena, Italia; Rivierenhof Park, Deurne, Belgium; Späth-Arboretum, Humboldt-Universität zu Berlin, Berlin, Germany.

Genus	Infrageneric group	Species	Ecological distribution	Seed origin*	Voucher number
*Quercus*	*Cerris*	*Q. acutissima* Carruth.	East of Asia	Vila Real, Portugal	HVR20134
		*Q. castaneifolia* C.A. Mey.	SW Asia	Vila Real, Portugal	HVR20135
		*Q. cerris* L.	Europe and SW Asia	Siena, Italy	HVR20136
		*Q. crenata* Lam. (*Q. cerris* × *Q. suber*)	Iberian Peninsula	Vila Real, Portugal	HVR20139
		*Q. libani* G. Oliver	E Medit. and Asia Minor	Berlin, Germany	HVR20658
		*Q. suber* L.	SW Europe and NW Africa	Vila Real, Portugal	HVR20153
	*Ilex*	*Q. coccifera* L.	Mediterranean	Vila Real, Portugal	HVR20137
		*Q. ilex* L.	Mediterranean	Müenster, Germany	HVR20141
		*Q. ilex* subsp. *rotundifolia* Lam.	Mediterranean	Vila Real, Portugal	HVR20142
		*Q. phillyraeoides* A. Gray	SW Asia	Kyoto, Japan	HVR20148
	*Lobatae*	*Q. coccinea* Muenchh.	North America	Vila Real, Portugal	HVR20138
		*Q. kelloggii* Newb.	California and SW Oregon	Lyon, France	HVR20143
		*Q. palustris* Münchh.	NE America	Berlin, Germany	HVR20145
		*Q. phellos* L.	NE America	Vila Real, Portugal	HVR20147
		*Q. rubra* L.	NE America	Hunedoara, Romania	HVR20152
		*Q. velutina* Lamb.	NE America	Berlin, Germany	HVR20657
	*Quercus*	*Q. faginea* Lam.	SW Europe	Vila Real, Portugal	HVR20666
		*Q. fraineto* Ten.	SE Europe and SW Asia	Vila Real, Portugal	HVR20140
		*Q. mongolica* Fisch. Ex Turcz.	East of Asia	Tübingen, Germany	HVR20144
		*Q. petraea* (Mattuschka) Liebl.	Europe and SW Asia	Tallinn, Estonia	HVR20146
		*Q. pubescens* Willd.	Europe and SW of Asia	Lyon, France	HVR20149
		*Q. pyrenaica* L.	SW Europe and N Africa	Bayreuth, Germany	HVR20150
		*Q. robur* L.	Europe and SW Asia	Riga, Latvia	HVR20151
		*Q. serrata* Thunb. ex Murray	East of Asia	Kanagawa, Japan	HVR20659
*Castanea*		*C. crenata* Siebold & Zucc.	Japan and South Korea	Kyoto, Japan	HVR20128
		*C. mollissima* Blume	China	Vila Real, Portugal	HVR20129
		*C. sativa* Mill.	Europe and SW Asia	Deurne, Belgium	HVR20130
*Fagus*		*F. japonica* Maxim.	Japan	Vila Real, Portugal	HVR20133
		*F. sylvatica* L.	Europe and SW Asia	Kaunas, Lithuania	HVR20131
		*F. sylvatica* (Atropurpurea Group) ‘Riversii’ AGM	Europe	Duisburg, Germany	HVR20132

Regarding the taxonomy of the genus *Quercus*, we adopted (except for *Q. serrata*) the informally unranked infrageneric ‘groups’ proposed by Denk and Grimm (2009, 2010). Thus, the oak species used in this study were classified as *Cerris* (cerris oaks; ecologically confined to Eurasia), *Ilex* (ilex oaks; Eurasia), *Quercus* (white oaks; Eurasia and North America) and *Lobatae* (red oaks; Americas). We assumed that the hybrid *Q. crenata* would belong to the *Cerris* group along with the parental *Q. cerris* and *Q. suber*. All the studied species have ecological origins that are representative of the natural distributions of the Fagaceae across the northern hemisphere.

To rule out hybridization issues between the mother trees of the plants used for DNA extraction, the seeds were harvested in different botanical gardens (Table 1), ensuring that the plants had morphological characters that enabled unambiguous classification. Dormancy was broken at 4 °C, and germination occurred at 7 °C in the dark on moistened cotton. Plantlets were grown in peat moss under greenhouse conditions at the University of Tras-os-Montes and Alto Douro (Vila Real, Portugal).

### Genomic DNA extraction and ITS amplification

To avoid fungal contamination in the ITS amplification, young, healthy leaves from one individual per species were collected and thoroughly cleaned with 70 % v/v alcohol and deionized water for further genomic DNA extraction using the cetyl trimethylammonium bromide method (Doyle and Doyle 1987). The integrity evaluation was done by electrophoresis performed in 0.8 % w/v agarose gels and by DNA quantification in a Nanodrop ND-1000 spectrophotometer. DNA stocks were diluted to a concentration of 50 ng μL^−1^.

The complete ITS region (ITS1-5.8S-ITS2) was amplified using the primers ITS-5 (5′-GGAAGTAAAAGTCGTAACAAGG-3′) as forward and ITS-4 (5′-TCCTCCGCTTATTGATATGC-3′) as reverse, both from White *et al.* (1990). These primers were designed for the highly conserved 5′-end of the 26S rRNA gene and for the 3′-end of the 18S rRNA gene, respectively (White *et al.* 1990). Each PCR (final volume of 50 µL) consisted of 50 ng of template DNA, 5μL KCl reaction buffer, 2.5 mM of each dNTP, 62 µM of each primer and 4 units of Dream Taq DNA polymerase (Thermo Fisher Scientific, Waltham, MA, USA). Ten microlitres of each amplified product were loaded on 1.5 % w/v agarose gels stained with ethidium bromide in order to confirm the ITS amplification.

### ITS PCR–RFLP markers

Nine restriction enzymes were tested for the production of ITS PCR–RFLP markers: AluI, BamHI, DpnI, EcoRI, HaeIII, HpaII, RsaI, Sau96I and TaqI (Thermo Fisher Scientific). The digestion reactions (final volume of 30 µL) followed the manufacturer's instructions. In each digestion reaction, 10 µL of the amplified product were used. The enzymatic reaction was thermally interrupted, and the digested products were separated by electrophoresis performed in 2 % w/v agarose gels stained with ethidium bromide. The sizes of the fragments were estimated by comparison with the molecular weight marker Gene Ruler DNA Ladder 100 bp Plus (Thermo Fisher Scientific) loaded in each agarose gel.

### Statistical analysis

Despite being co-dominant markers, the ITS fragments were scored as presence (1) or absence (0) in order to construct binary matrices for statistical analysis. For each genus and the *Quercus* infrageneric groups, the software POPGENE 1.32 (Yeh *et al.* 1999) was used to calculate Nei's expected heterozygosity (*H*_E_; Nei 1973) and Shannon's information index (*I*; Lewontin 1972) to measure the level of gene diversity, and the same software was used to estimate Nei's original measures of genetic identity and genetic distance (Nei 1973). To evaluate the phylogenetic relationships, a radial tree and a phylogram were generated with TreeView (Page 1996) based on the genetic distance (Nei 1987) and neighbour-joining method (Saitou and Nei 1987), calculated using POPGENE. A pairwise similarity matrix was calculated using Dice's coefficient, and a dendrogram of genetic similarity was constructed using the unweighted pair group method with arithmetical averages (UPGMA) and the Numerical Taxonomy and Multivariate Analysis System software (NTSYS-PC 2.02) (Rohlf 1998). A bootstrap analysis based on 1000 replications was performed with Winboot (Yap and Nelson 1996) to test the clustering reliability. Only bootstrap values >50 % and common to the consensus trees generated either by Winboot and UPGMA are shown (Felsenstein 1985). The adjustment fitness of the UPGMA dendrogram to the binary matrices was verified using the COPH and MXCOMP modules from the NTSYS software. To certify the NTSYS UPGMA clustering and estimate the genetic structure of the individuals under study, we used STRUCTURE 2.3.4 software (Pritchard *et al.* 2000), where several clusters (*K*) were tested with 50 000 generations of burn-in and 100 000 Markov chain Monte Carlo iterations.

## Results and Discussion

### ITS amplification

The ITS1-5.8S-ITS2 length usually varies from 500 to 750 bp in angiosperms (Baldwin *et al.* 1995). Phylogenetic studies based on ITS sequencing often detect, beyond point mutations, short nucleotide insertions/deletions responsible for the variable length of the ITS1-5.8S-ITS2 region. Such an event is related to dissimilarities in the spacers, since the 5.8S gene exhibits high sequence conservation and minimal variation in the length of ∼163−165 bp in several species (Baldwin *et al.* 1995; Manos *et al.* 2001; Denk *et al.* 2002; Papini *et al.* 2011).

Regarding the results in the genus *Quercus*, a single band of ∼700 bp was amplified in 19 species (data not shown). However, in five oak species, two ITS bands of ∼600 and 700 bp in length were detected. In *Q. cerris* and *Q. fraineto*, the two bands exhibited the same intensity in the agarose gel, while in the other three species (*Q. crenata*, *Q. castaneifolia* and *Q. ilex* subsp. *rotundifolia*), the 700-bp band was more intense. Several authors have reported variable ITS lengths for the same oak species. Some examples are described below, and may be attributed to different amplification conditions or primers. Manos *et al.* (2001) reported an alignment of 635 bp for 179 Fagaceae taxa. Muir *et al.* (2001) detected a single band of ∼720 bp in *Q. petraea* and *Q. robur*. Bellarosa *et al.* (2005) amplified a single band of ∼600 bp in 11 oak species (some common to this study: *Q. cerris*, *Q. suber*, *Q. fraineto*, *Q. petraea*, *Q. pubescens*, *Q. robur*, *Q. coccifera* and *Q. ilex*). Casas *et al.* (2007) reported an ITS length ranging from 599 to 607 bp in six oak species (*Q. pubescens*, *Q. faginea*, *Q. pyrenaica*, *Q. suber*, *Q. ilex* and *Q. coccifera*). Regarding the detailed lengths, Manos *et al.* (1999) assumed a size of 614 bp in 44 *Quercus* species after detecting different lengths for ITS1 (223−234 bp) and ITS2 (206−214 bp), while 5.8S exhibited a rather stable length of 164 bp. Papini *et al.* (2011) reported a 615-bp matrix (indels included), where ITS1 represented 212 bp in *Q. iberica* and 222 bp in *Q. macranthera*, while the ITS2 and the 5.8S coding region were 211 and 163 bp, respectively, in both species.

The three *Castanea* species showed bands ∼600 bp in length, but an additional band of ∼700 bp was amplified in *Castanea mollissima* and *C. sativa* (data not shown). Interestingly, the 600-bp band was more intense in *C. mollissima*, and the 700-bp band was more intense in *C. sativa*. According to Samuel *et al.* (1998), the lengths in *Castanea* are 231 bp for ITS1 (U93097) and 183 bp for ITS2 (U93098).

Denk and Grimm (2010) suggested that the dissimilarities in *Fagus* ITS1 to other Fagaceae may discourage comparisons in phylogenetic studies. Indeed, Manos *et al.* (2001) found a 635-bp alignment for 179 Fagaceae taxa, but, in *Fagus*, an additional 40-bp insertion occurred in ITS1. Despite this, we decided to include our results in the pooled data to understand how they would stand out from the remaining genera and to survey for intra-specific differences. In the three *Fagus* species studied here, a single band ∼750 bp long was amplified (data not shown), confirming its longer length. Denk *et al.* (2002) encountered a total length varying from 644 to 660 bp, average lengths of 259 bp for ITS1, 229 bp for ITS2 and a stable length of 165 bp for the 5.8S gene.

**Figure 1.**
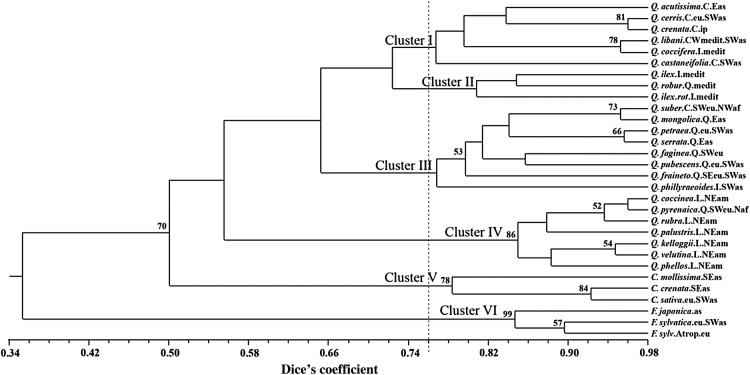
Unweighted pair group method with arithmetical averages dendrogram of genetic similarity based on the pool of ITS PCR–RFLP data achieved with the restriction enzymes HaeIII, HpaII, Sau96I and TaqI. Only bootstrap values >50 are represented. The ecological distribution is indicated after species name (am, North America; as, Asia; eu, Europe; medit, Mediterranean; and af, Africa), and the infrageneric group for each *Quercus* species (C, *Cerris*; I, *Ilex*; L, *Lobatae*; and Q, *Quercus*).

The ITS length variants and ITS sequence variants (amplified from different loci) can be found in one organism. The former diverge in size due to insertion or deletion events, and the latter (while having the same length) exhibit sequence polymorphisms. The fact that length variants were amplified in five oaks and two chestnuts suggests either intra-individual rDNA polymorphism (due to the lack of homogenization within rDNA paralogues) or amplification of pseudogenes (non-functional paralogues that are transcriptionally inactive). Also, it should not be necessary to characterize all the arrays of a genome, because concerted evolution means that (i) gel results will be leveraged by the more prevalent length in the rDNA pool and (ii) sequencing results may be based on cloned fragments, where similarity is expected. Harpke and Peterson (2008) suggested that pseudogenes could be identified by searching for substitutions in the 5.8S sequence, but since our study on the ITS variability relied on an RFLP analysis, such an approach was not possible. Therefore, despite the fact that both ITS variants could occur, the clustering of these seven species was relatively precise, and the results are consistent with the adopted taxonomy (Table 1, Fig. 1). On this subject, Saini *et al.* (2008) also found two ITS length variants in the genus *Vigna* and stated that the ITS variants, when analysed together, did not cause any phylogenetic errors at the species level. Taking these considerations into account, we digested all the ITS amplicons to produce ITS PCR–RFLP markers.

### ITS PCR–RFLP data

For a preliminary assessment of ITS polymorphism, nine restriction enzymes were tested in *Q. petraea*: AluI, BamHI, DpnI, EcoRI, HaeIII, HpaII, RsaI, Sau96I and TaqI. The four enzymes that provided the most clearly visible and polymorphic restriction fragments were selected: HaeIII (5′-GG↓CC-3′), HpaII (C↓CGG), Sau96I (G↓GNCC) and TaqI (T↓CGA).

The molecular sizes of the restriction fragments are indicated in Table 2. The four selected restriction enzymes revealed polymorphisms among the 30 species. No specific pattern was observed according to *Quercus* infrageneric group, but all enzymes produced genus- and species-specific ITS PCR–RFLP patterns. The four restriction enzymes produced a total of 50 ITS PCR–RFLP patterns (Table 2), and several bands were common among patterns (Table 3). Each restriction enzyme gave rise to 12−13 patterns that occurred in a particular genus, group and/or species. Some patterns were shared among different taxa. Despite two patterns being present in all *Lobatae* species (Sau96I and TaqI), they could not be considered group-specific because they were shared with *Q. pyrenaica*. With the exception of TaqI, the other patterns of *Q. coccifera* were shared with oaks from several groups. In *Q. robur*, species-specific patterns were revealed for HaeIII and HpaII; for Sau96I and TaqI, shared patterns were observed in *Q. ilex* and *Q. ilex* subsp. *rotundifolia*. TaqI also produced a pattern specific to the genus *Fagus*. Again with the exception of TaqI in the *Cerris*, *Ilex* and *Quercus* groups, the other three enzymes produced monomorphic bands in the three genera and in all *Quercus* infrageneric groups (Table 3). An ITS PCR–RFLP independent analysis of the six taxonomic levels per restriction enzyme revealed levels of polymorphism that ranged from 0 to 100 %, and unique bands were found only for *Fagus* (Table 3).

**Table 2.** ITS PCR–RFLP patterns achieved with each restriction enzyme (RE) among the studied Fagaceae species (*Quercus* infrageneric groups in parentheses).

RE pattern	Bands (bp)	Species with the same ITS PCR–RFLP pattern
HaeIII		
* *a	180, 225, 415, 480	*Q. rubra* (*L*); *Q. mongolica* (*Q*); *Q. pyrenaica* (*Q*)
* *b	180, 225	*Q. acutissima* (*C*); *Q. castaneifolia* (*C*); *Q. libani* (*C*); *Q coccifera* (*I*); *Q. ilex* (*I*); *Q. phillyraeoides* (*I*); *Q. kelloggii* (*L*); *Q. velutina* (*L*); *Q. serrata* (*Q*)
* *c	155, 480, 620	*C. crenata*
* *d	100, 120, 180, 210, 290	*F. japonica*
* *e	180, 225, 260, 415, 480	*Q. coccinea* (*L*)
* *f	155, 180, 225, 260, 480	*Q. ilex* subsp. *rot*. (*I*)
* *g	180, 225, 260, 415, 460	*Q. palustris* (*L*)
* *h	180, 225, 380, 460	*Q. phellos* (*L*)
* *i	180, 225, 315, 430, 480	*Q. robur* (*Q*)
* *j	155, 180, 225, 480	*C. mollissima*, *C. sativa*
* *k	100, 120, 180, 210, 460	*F. sylvatica*, *F. sylvatica Atropurpurea*
* *l	180, 225, 480	*Q. cerris* (*C*); *Q. crenata* (*C*); *Q. suber* (*C*)
* *m	180, 225, 460	*Q. faginea* (*Q*); *Q. fraineto* (*Q*); *Q petraea* (*Q*); *Q. pubescens* (*Q*)
HpaII		
* *a	185, 250	*Q. acutissima* (*C*); *Q. kelloggii* (*L*); *Q. rubra* (*L*)
* *b	185, 220, 250, 470	*Q. libani* (*C*); *Q. coccifera* (*I*); *Q. petraea* (*Q*); *Q. serrata* (*Q*)
* *c	185, 220, 250	*Q. cerris* (*C*); *Q. crenata* (*C*); *Q. suber* (*C*); *Q. ilex* (*I*); *Q. fraineto* (*Q*); *Q. mongolica* (*Q*)
* *d	185, 250, 470	*Q. pubescens* (*Q*); *Q. pyrenaica* (*Q*); *Q. coccinea* (*L*); *Q. palustris* (*L*); *Q. phellos* (*L*); *Q. velutina* (*L*)
* *e	140, 185, 270, 470	*C. mollissima*
* *f	185, 250, 430, 470	*Q. castaneifolia* (*C*)
* *g	185, 220, 250, 430	*Q. faginea* (*Q*)
* *h	185, 220, 250, 270	*Q. ilex* subsp. *rot*. (*I*)
* *i	140, 185, 220, 250, 400	*Q. phillyraeoides* (*I*)
* *j	185, 220, 250, 330, 470	*Q. robur* (*Q*)
* *k	140, 185, 270	*C. crenata*, *C. sativa*
* *l	220, 470	Genus *Fagus* (all species)
Sau96I		
* *a	100, 150, 350, 490	*Q. ilex* (*I*); *Q. ilex* subsp. *rot. (I*); *Q. robur* (*Q*)
* *b	100, 150, 490	*Q. phillyraeoides* (*I*); *Q. petraea* (*Q*); *Q. serrata* (*Q*)
* *c	150, 490	*Q. acutissima* (*C*); *Q. libani* (*C*); *Q. suber* (*C*); *Q. coccifera* (*I*); *Q. faginea* (*Q*); *Q. mongolica* (*Q*); *Q. pubescens* (*Q*)
* *d	215, 490	Group *Lobatae* (all six species); *Q. pyrenaica* (*Q*)
* *e	150, 170, 490	*C. mollissima*
* *f	100, 150, 450, 560, 690	*F. japonica*
* *g	100, 150, 560, 650	*F. sylvatica*
* *h	100, 150, 450, 490, 560	*F. sylvatica Atropurpurea*
* *i	100, 150, 390, 490, 650	*Q. castaneifolia* (*C*)
* *j	150, 490, 650	*Q. cerris* (*C*)
* *k	100, 150, 490, 650	*Q. crenata* (*C*)
* *l	100, 150, 170, 490	*Q. fraineto* (*Q*)
* *m	150, 490	*C. crenata*, *C. sativa*
TaqI		
* *a	115, 145, 200, 265, 350	*Q. ilex* (*I*); *Q. robur* (*Q*)
* *b	265, 350	*Q. suber* (C); *Q. faginea* (*Q*); *Q. mongolica* (*Q*); *Q. petraea* (*Q*); *Q. pubescens* (*Q*); *Q. serrata* (*Q*)
* *c	115, 145, 250	Group *Lobatae* (all six species); *Q. pyrenaica* (*Q*)
* *d	145, 265	*C. crenata*
* *e	145, 200, 265	*Q. coccifera* (*I*)
* *f	115, 265, 350	*Q. fraineto* (*Q*)
* g*	115, 145, 200, 350	*Q. ilex* subsp. *rot*.* * (*I*)
* *h	145, 200	*Q. libani* (*C*)
* *i	225, 265, 350	*Q. phillyraeoides* (*I*)
* *j	145, 200, 265	*C. mollissima*, *C. sativa*
* *k	145, 265, 375	Genus *Fagus* (all three species)
* *l	115, 145, 200	*Q. acutissima* (C); *Q. castaneifolia* (C); *Q. cerris* (C); *Q. crenata* (*C*)

**Table 3.** An individual analysis was performed in *Castanea* and *Fagus* genera, and each *Quercus* infrageneric group (*Cerris*, *Ilex*, *Lobatae* and *Quercus*) for the size (bp) of the monomorphic (*M*) and polymorphic (*P*) bands, and the polymorphism percentage (%*P*) detected per RE. The unique (U) bands were evaluated among the six studied taxa.

RE	Taxa	*M* (bp)	*P* (bp)	%*P*	*U* (bp)
HaeIII	*Castanea*	155, 480	180, 225, 620	60.0	None
	*Fagus*	100, 120, 180, 210	290, 460	33.3	100, 120, 210
	*Cerris*	180, 225	480	33.3	None
	*Ilex*	180, 225	155, 260, 480	60.0	None
	*Lobatae*	180, 225	260, 380, 415, 460, 480	71.4	None
	*Quercus*	180, 225	315, 415, 430, 460, 480	71.4	None
HpaII	*Castanea*	140, 185, 270	470	25.0	None
	*Fagus*	220, 470	None	00.0	None
	*Cerris*	185, 250	220, 430, 470	60.0	None
	*Ilex*	185, 220 , 250	140, 270, 400, 470	57.1	None
	*Lobatae*	185, 250	470	33.3	None
	*Quercus*	185, 250	220, 330, 430, 470	66.7	None
Sau*9*6I	*Castanea*	150, 490	170	33.3	None
	*Fagus*	100, 150, 560	450, 490, 650, 690	57.1	560
	*Cerris*	150, 490	100, 390, 650	60.0	None
	*Ilex*	150, 490	100, 350	50.0	None
	*Lobatae*	215, 490	None	00.0	None
	*Quercus*	490	100, 150, 170, 215, 350	83.3	None
TaqI	*Castanea*	145, 260	200	33.3	None
	*Fagus*	145, 260, 375	None	00.0	375
	*Cerris*	None	115, 145, 200, 265, 350	100	None
	*Ilex*	None	115, 145, 200, 225, 265, 350	100	None
	*Lobatae*	115, 145, 250	None	00.0	None
	*Quercus*	None	115, 145, 200, 250, 265, 350	00.0	None

Whenever the RFLP assay returns bands whose summed lengths exceed the uncut product, the presence of dissimilar loci in a genome is implied, reflecting the existence of sequence variants. In fact, Redecker *et al.* (1997) suggested that when the restriction patterns indicate the presence of two sets of sequences, because the overall length of the fragments is much larger than the original, uncut PCR product, this must be attributed to sequence heterogeneity.

When considering the ITS PCR–RFLP data observed among all Fagaceae individuals and all restriction enzymes, 100 % polymorphism was observed (Table 4, first row). The rates of polymorphism ranged from 33.3 % in *Fagus* taxa to 100 % in Fagaceae taxa. A high number of phenotypes (12 or 13) were identified per restriction enzyme, which corroborates the medium levels of polymorphism observed.

**Table 4.** Genetic diversity analyses based on the pool of the RFLP data produced with the four REs, performed in *Castanea* and *Fagus* genera, and each *Quercus* infrageneric group (*Cerris*, *Ilex*, *Lobatae* and *Quercus*): total number of RFLP bands (*T*); number of polymorphic bands (*P*); percentage of polymorphism (%*P*); Nei's expected heterozygosity (*H*_E_, Nei 1973); and Shannon's information index (*I*).

Taxa	*T*	*P*	%*P*	*H*_E_	*I*
Fagaceae	43	43	100	0.2031	0.3297
*Castanea*	15	6	40.0	0.1575	0.2316
*Fagus*	18	6	33.3	0.1208	0.1816
*Quercus*	34	29	85.3	0.1753	0.2829
*Cerris*	18	12	66.7	0.2226	0.3367
*Ilex*	22	15	68.2	0.2357	0.3557
*Lobatae*	15	6	40.0	0.1307	0.2008
*Quercus*	25	20	80.0	0.2162	0.3406

The expected heterozygosity (*H*_E_) was 0.2031 in the family Fagaceae (Table 4), with averages of 0.1512 among the three genera and 0.2013 among the four *Quercus* infrageneric groups (calculations from Table 4 data). The total diversity of the patterns, measured using Shannon's diversity index (*I*), was 0.3297. Despite the low values exhibited for all taxa, Shannon's diversity index varied according to the *H*_E_. In the *Quercus* infrageneric groups, this index was similar in *Cerris*, *Ilex* and *Quercus* (∼0.34). The much lower value in *Lobatae* (0.2008) was probably due to the geographic isolation of the American oaks. Overall, such relationships were reflected in the dendrogram (Fig. 1). These genetic diversity results are also consistent with those presented by Quijada *et al.* (1998) and Carvalho *et al.* (2009) using the same molecular marker technique with some of the restriction enzymes used here. Such low levels of heterozygosity might be due to the co-dominant nature of these markers.

The pool of the ITS PCR–RFLP data was used to construct an UPGMA dendrogram of genetic similarity among the 30 Fagaceae species under study in order to estimate their phylogenetic relationships based on these alternative co-dominant markers (Fig. 1). The high correlation coefficient (*r* = 0.89) ensured that the UPGMA clustering of the 30 Fagaceae species is an accurate representation of the data matrix, with 36 % total genetic similarity. Considering a cut-off value of 0.76, the dendrogram may be divided into six major clusters that correspond to each one of the four *Quercus* infrageneric groups (*Cerris* [Cluster I], *Ilex* [Cluster II], *Quercus* [Cluster III] and *Lobatae* [Cluster IV]) and to *Fagus* (Cluster V) and *Castanea* (Cluster VI) genera (Fig. 1). The genetic similarity among the 24 *Quercus* species was 0.57, confirming the moderate ITS PCR–RFLP polymorphisms visualized on the agarose gels (data not shown). The bootstrap analysis also corroborated several clusters at different taxonomic levels (Fig. 1). Although correctly clustered in the *Quercus* genus along with the other 19 oaks, five oaks (*Q. coccifera*, *Q. phillyraeoides*, *Q. pyrenaica*, *Q. robur*, and *Q. suber*) were not clustered according to the taxonomic infrageneric group. All of these oaks amplified a unique ITS1-5.8-ITS2 band and clustered among others with similar distribution areas, suggesting that such ITS PCR–RFLP resemblances could be due to the sharing of earlier ancestors.

Cluster I included oaks from the *Cerris* group, with only *Q. suber* excluded. Hybrids (particularly those occurring in the overlapping limits of the species distribution areas) tend to coexist with the parental species if these new intermediate genotypes have characters more suitable for the environmental circumstances. In fact, a genetic similarity of 96 % was found between *Q. crenata* and the parent *Q. cerris*, with only 66 % similarity between *Q. suber* and the former two. Bellarosa *et al.* (2005) reported a contradictory result, with higher proximity between *Q. crenata* and *Q. suber*. Such a circumstance may be attributed to the molecular markers' potential for the fingerprinting of natural hybrids. With respect to the uniparental inherited cpDNA, Muir and Schlötterer (2006) suggested that some areas of the hybrid genome could escape from selection towards species integrity. Despite the biparental inheritance of rDNA, homogenization of the entire rDNA arrays towards one parental type could take place in hybrids. Our results were similar to those of Conte *et al.* (2007), who emphasized that despite being auto-allogamous, ‘asymmetrical backcrossing’ of *Q. crenata* in a mixed population could lead the hybrid progeny towards the predominant parental species. Although in a different context, the hypothesis of ‘directed’ introgression was also noted by Casas *et al.* (2007) for the difficulties encountered in finding ‘morphologically identifiable hybrids' between *Q. coccifera* and other *Quercus* species (except for *Q. ilex*). In our work, *Q. coccifera* (group *Ilex*) was included in Cluster I, separately from the other oaks of the same group. Taxonomically, this was a surprising clustering. It was also surprising in the molecular sense, since the large number of cpDNA haplotypes shared between *Q. ilex* and *Q. coccifera* led Jiménez *et al.* (2004) to suggest that both species might experience incomplete lineage sorting or hybridization. Denk and Grimm (2010) found ‘zero distance’ based on complete ITS sequencing, and Coutinho *et al.* (2014) found significant genetic similarity between *Q. ilex* and *Q. coccifera* using inter simple sequence repeats (ISSRs). If *Q. coccifera* clustered near *Q. libani* (Group *Cerris*), and since their distribution overlaps in the Mediterranean region, it is possible that both rDNAs experienced the same evolutionary pressures during speciation. In short, even if not in total agreement with the adopted taxonomy, both Clusters I and II are supported by the sharing of morphological (not homoplastic) and ecological features between the *Cerris* and *Ilex* groups.

Cluster II included two species from the *Ilex* group, *Q. ilex* and *Q. ilex* subsp. *rotundifolia*, and *Q. robur* from the *Quercus* group, with the three species exhibiting a Mediterranean-based distribution. The proximity between *Q. ilex* and *Q. ilex* subsp. *rotundifolia* may be due to rDNA array homogenization that preceded the speciation event. Nonetheless, *Q. ilex* and *Q. robur* share the natural hybrid *Q. turneri* Willd. A slow ‘molecular drive’ (Dover 1982, 1986) could explain such proximity; being Mediterranean species, both might have experienced similar adaptive conditions. Mutations, insertions or deletions are the changes in the recognition sites needed to explain the presence in this cluster of *Q. robur* (*Quercus* group), the oak-type of the ‘roburoid oaks’.

Constituting a single branch in Cluster III, the *Q. phillyraeoides* (*Ilex* group) only shares a Southwest Asia distribution with the *Quercus* group oaks. In a previous study using ISSRs, this species was clustered in the *Ilex g*roup (Coutinho *et al.* 2014). *Quercus phillyraeoides* exhibited two specific patterns revealed by the HpaII and TaqI enzymes, and shared patterns (produced by HaeIII) with two oaks from the *Quercus* group that dictated its inclusion in this cluster. The remaining species included in Cluster III are oaks from the *Quercus* group, with the exception of *Q. suber* (*Cerris* group). Similar clustering was recently found based on ISSRs (Coutinho *et al.* 2014). *Quercus suber* could have originated in the Middle East (Bellarosa *et al.* 2005 and references therein) and expanded westward (Lumaret *et al.* 2005). This feature could somehow explain its clustering along with oaks nowadays distributed throughout Eurasia. Regarding the inter-fertile *Q. robur* and *Q. petraea* (the two predominant white oaks in deciduous European forests), Muir *et al.* (2001) could not differentiate these based on ITS sequencing, and suggested that the split between the two may be too recent for rDNA to have diverged. Nevertheless, despite being in different clusters, the existing differences in the recognition sites allowed the ITS-RFLP markers to distinguish the two species. *Quercus serrata* was not included in the studies of Denk and Grimm (2009, 2010) on the infrageneric classification of the *Quercus* genus. In our work, it clustered along with oaks from the *Quercus* group. This broad-leaved deciduous oak is widespread in the forests of eastern Asia, and was classified as belonging to the *Lepidobalanus* section (Camus 1938–39) along with the following: *Q. faginea* (subsection *Gallifera*); *Q. petraea* and *Q. pubescens* (both subsection *Sessiliflorae*); *Q. robur* (subsection *Pedunculatae*); and *Q. ilex* (subsection *Ilex*). With the exception of *Q. ilex* (*Ilex* group), all these species were classified as the infrageneric *Quercus* group (based on the taxonomy adopted for this work). In this sense, we included this oak in the *Quercus* group and interpreted the dendrogram result as adequate clustering.

All the American oaks (*Lobatae* group) were grouped in Cluster IV, suggesting that their geographic isolation led the ITS rDNA region in a different evolutionary direction from the European or Asian oaks. Even *Q. kelloggii*, which exhibits morphological features distinct from all other lobed-leaf eastern oak species (Nixon 2002), was clustered accordingly. Strangely, the Southern European *Q. pyrenaica* (*Quercus* group) shares four RFLP patterns with North American oaks, and thus appears in this cluster. The molecular similarity in the ITS sequences of some European and North American oaks from the *Quercus* group (Denk and Grimm 2010) and the pollen characteristics found in fossil sediments have highlighted the importance of Iceland as a ‘North Atlantic land bridge’ before and even after the intercontinental disjunctions of northern temperate trees (Denk *et al.* 2010). Also, oak migrations between North America and East Asia via the Bering land bridge were suggested due to the presence, in East Asia and Europe, of leaves and pollen fossil records with characteristics similar to the *Lobatae* group oaks (Denk *et al.* 2012). Nevertheless, despite depending only on short recognition sites, the ITS PCR–RFLP clustered all the American oaks in a single cluster. The inclusion of *Q. pyrenaica* could be due to mutations in these four or five nucleotide sequences, but it was remarkable that all patterns were shared with American oaks.

The species from the *Castanea* and *Fagus* genera were grouped in independent clusters (Fig. 1). The three *Castanea* species belong to the *Eucastanon* section and constitute Cluster V. High similarity was detected between the Chinese and the European chestnuts, resembling the results achieved by Lang *et al.* (2006, 2007). Those authors suggested that the Japanese *Castanea crenata* holds a basal position in the genus phylogeny, and that Eastern Asia could have constituted the centre of diversification towards the east (China and Japan), followed later by the invasion heading to the west, promoting Chinese and European species divergence.

The three beeches studied here from the species *Fagus japonica* and *F. sylvatica*, belonging to the *Engleriana* and *Fagus* subgenera, respectively, were clustered in Cluster VI and showed 15 % genetic dissimilarity (Fig. 1). As expected, the genetic similarity was higher between the two *F. sylvatica* species than among those and *F. japonica*. The ITS PCR–RFLP data revealed 34 % of genetic similarity among the *Fagus* species and the remaining Fagaceae. Interestingly, the major differences in the ITS regions among *Fagus* species are located within the shorter ITS2 region (despite the relatively higher GC content that could promote higher sequence stability) (Denk *et al.* 2002), while the major differences between the *Fagus* genus and other Fagaceae are located in the larger ITS1 (Manos *et al.* 2001; Denk and Grimm 2010).

### Genetic structure

**Figure 2.**
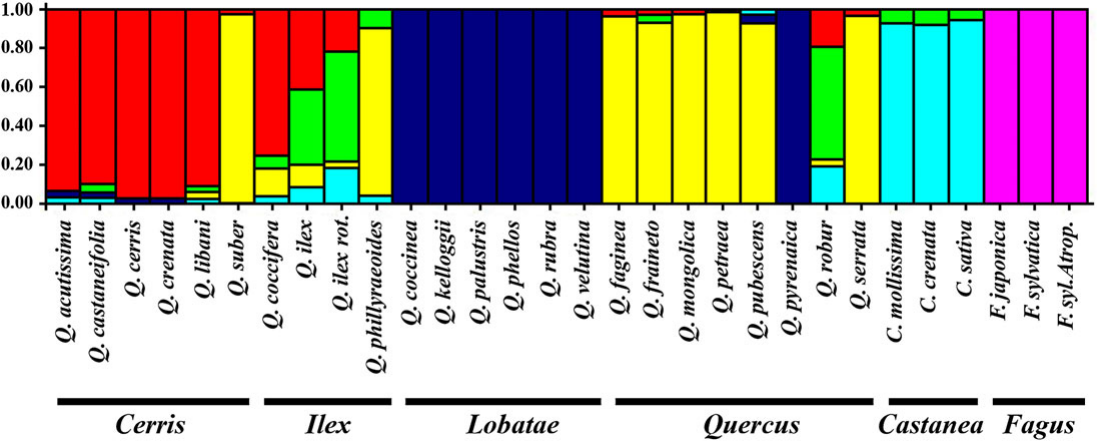
Bar plot constructed with the software STRUCTURE based on the pool of ITS PCR–RFLP data and inferred by Bayesian clustering analysis. Each coloured segment corresponds to the proportion of individuals assigned to a hypothetical population or cluster. Successive preset *K* values were calculated under the admixture model and the option of correlated allele frequencies, allowing the degree of admixture alpha to be inferred from the data. Only the *K* = 6 clustering (sorted by the original order) is shown, once it was determined as the most likely according to Pritchard *et al*. (2010).

The use of STRUCTURE 2.3.4 software (Pritchard *et al.* 2000) allowed the visualization of the genetic structure of the plant material used in this study (Fig. 2). The admixture model was used to evaluate the relationships between the sections of their genomes since they may have derived from different taxa. The optimal number (*K* = 6 clusters; Fig. 2) agreed with the UPGMA clustering (Fig. 1).

The genetic structure analysis provided fixation index (*F*_ST_) values that suggested high levels of differentiation (>0.50) for five clusters, corresponding to the *Castanea* genus (0.59), *Fagus* genus (0.61), *Cerris* group (0.51), *Lobatae* group (0.76) and *Quercus* group (0.5862). The sixth cluster, corresponding to the infrageneric *Ilex* group, revealed a reduced genetic differentiation value (*F*_ST_ = 0.26), sustaining the dispersion of these oaks through the dendrogram. The bar plot showed that four oaks of the *Ilex* group and one oak (*Q. robur*) of the *Quercus* group seemed to share some ancient genetic material with the *Castanea* genus. It is assumed that both *Quercus* and *Castanea* genera share an ancient origin, and the fact that these oaks have a narrow distribution area might have contributed to the conservation of such similarities and the lack of higher genetic fixation (as observed for the other species/groups). In the bar plot, it is apparent that three out of four *Ilex* individuals share material with the *Cerris* group, confirming the 0.74 genetic similarity between these groups found in the dendrogram (Fig. 1). The high *F*_ST_ value for *Fagus* species suggested a defined ITS genetic structure and confirmed the ITS divergence from the other taxa. In the dendrogram, *Q. phillyraeoides* and *Q. suber* were clustered along with oaks of the *Quercus* group, and the bar plot suggested their high similarity. The same occurred between *Q. pyrenaica* (*Quercus* group) and oaks from the *Lobatae* group. Also, the *Lobatae* group was found to be the most genetically fixed, which could be explained by their ancient isolation in North America.

The genetic identities and genetic distances (calculated using POPGENE and the ITS PCR–RFLP pool data, data not shown) of the three genera and four *Quercus* infrageneric groups supported most of these results. *Quercus* and *Castanea* genera were evaluated to be more intimately related to each other (0.82) than to *Fagus* (0.69 and 0.64, respectively). The genetic relations in the infrageneric groups seem to resemble their geographical distributions: *Lobatae* exhibited the highest genetic distance relative to *Cerris*, *Ilex* and *Quercus* (0.153, 0.224 and 0.171, respectively); *Cerris* and *Ilex* were the most related (0.962); and *Quercus* revealed similar proximities to both *Cerris* (0.947) and *Ilex* (0.960).

## Conclusions

This study of ITS genotaxonomy intended to evaluate the potential of the ITS-PCR–RFLP markers for inferences about the Fagaceae family taxonomy, particularly in the *Quercus* genus. In phylogenetics, given the stability of the sequence of the 5.8S rRNA gene, the main use of the ITS1-5.8S-ITS2 region has been for comparing the sequence of the two spacers. When using restriction enzymes, in which accuracy resides in short recognition sites defined by four or five nucleotides, even a single-nucleotide mutational event may constrain the final result. Nonetheless, the conservation of those enzyme cutting sites in closely related species may allow the use of RFLP patterns to make inferences about phylogenies. Indeed, according to the adopted taxonomy, correct clustering was observed for (i) the 30 individuals per genus, (ii) 19 of the 24 oaks per *Quercus* infrageneric group (almost 80 % accuracy) and (iii) 22 oaks per ecological area of distribution (>91 % accuracy). One must assume that site variability may distort the phylogenetic analysis, but our results and the advantages of cost-effectiveness, time consumption and higher discriminative power (especially between species with morphological similarities) suggest the effectiveness and utility of these markers for taxonomic discrimination in Fagaceae.

## Sources of Funding

This work was supported by the PhD grant SFRH/BD/42837/2008 awarded by the Portuguese Foundation for Science and Technology (FCT) and, partially, by the Institute of Biotechnology and Bioengineering—Centre of Genomics and Biotechnology of the University of Tras-os-Montes and Alto Douro IBB-CGB/UTAD.

## Contributions by the Authors

J.P.C. performed the practical work at the laboratory and analysed the results; J.P.C., A.C. and J.L.-B. contributed to the analysis and discussion of the results and to the writing of the manuscript.

## Conflicts of Interest Statement

None declared.
